# A Mini Literature Review of Probiotics: Transforming Gastrointestinal Health Through Evidence-Based Insights

**DOI:** 10.7759/cureus.57055

**Published:** 2024-03-27

**Authors:** Cara Mohammed, Jhon Philip Fuego, Karina V Garcia, Hira Jamil, Rahul Y Rajesh, Andres S Escobar, Muhammad J Hassan, Manju Rai

**Affiliations:** 1 Orthopedics, East Regional Health Authority, Port of Spain, TTO; 2 Internal Medicine, West Visayas State University College of Medicine, Iloilo City, PHL; 3 Internal Medicine, National Autonomous University of Mexico, Mexico City, MEX; 4 Medicine, University Medical and Dental College Faisalabad, Faisalabad, PAK; 5 Internal Medicine, Tbilisi State Medical University, Tbilisi, GEO; 6 Internal Medicine, Pontificia Universidad Javeriana, Cali, COL; 7 Internal Medicine, Faisalabad Medical University, Faisalabad, PAK; 8 Immunology, Shri Venkateshwara University, Gajraula, IND

**Keywords:** irritable bowel syndrome, diarrhea, constipation, colorectal cancer, ulcerative colitis, gastrointestinal diseases, dysbiosis, gut microbiota, probiotics

## Abstract

As our understanding of the intricate interaction between gut bacteria and human health continues to expand, so too has interest in the ability of probiotics to manage gut microbiota and confer multiple health benefits to the host. The mini literature review focuses on the expanding potential of the use of probiotics in GI health, with a focus on probiotics' potential therapeutic advantages in a variety of gastrointestinal (GI) illnesses. Probiotics play a significant role in managing diarrhea and symptoms of irritable bowel syndrome with diarrhea (IBS-D) by modulating gut microbial communities. Specific probiotic strains have been found to reduce the abundance of harmful bacteria, regulate inflammatory markers like interleukin 6, and improve GI symptoms such as abdominal discomfort and stool consistency. Additionally, probiotic blends have shown potential for preventing GI infections and alleviating GI pain in IBS-D patients. Studies have demonstrated that certain multi-strain probiotics, including *Bifidobacterium* and *Lactobacillus* species, can significantly increase the frequency of bowel movements and reduce the proportion of individuals experiencing constipation. It has also been found that probiotic supplementation may reduce the incidence of postoperative complications and mortality, particularly in patients undergoing colorectal adenocarcinoma surgery. Additionally, probiotics have been associated with decreased levels of pro-inflammatory cytokines and improved clinical outcomes in patients with colorectal cancer. Furthermore, probiotics have been associated with enhanced digestive tolerance, reduced GI inflammation, and prolonged clinical remission in certain UC patients. Studies have also shown that probiotics, administered either directly to infants or pregnant women during the perinatal stage, can alleviate symptoms such as inconsolable crying and irritation associated with infant colic, improve bowel movement frequency in cases of functional constipation, and enhance overall conditions in premature infants, including reducing regurgitation and improving feeding tolerance. The review addresses both encouraging results and challenges with probiotic therapy, while also arguing for more studies to elucidate underlying mechanisms and enhance therapeutic techniques. As we traverse the complex field of probiotic therapy in the treatment of GI illnesses, researchers, physicians, and other healthcare professionals can benefit from the informative information provided by this study.

## Introduction and background

**Figure 1:**
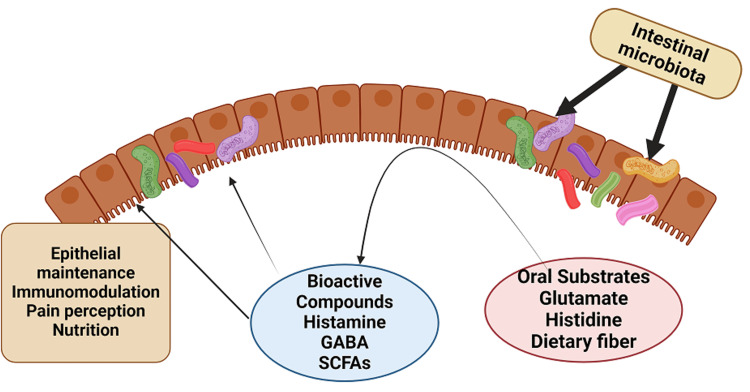
Intestinal microbes potentially play a vital role in host-microbiota interactions through luminal conversion Nutrients ingested orally can be metabolized by intestinal microbes into bioactive compounds, influencing both host health and the intestinal microbiota. Key examples include gamma-aminobutyric acid (GABA) and short-chain fatty acids (SCFAs). Image Credits: Created With Biorender.com

"Live microorganisms that, when administered in adequate amounts, confer a health benefit on the host organisms" is how the Food and Agriculture Organization and the World Health Organization describe probiotics [1]. Probiotics are frequently conceived of as dietary components that affect the microbiota of the human gut and have a regulating influence on the pattern and composition of the flora of the intestines. Through maintaining the mucosal barrier, supplying nutrients, and fostering disease resistance, intestinal flora directly impacts people's lives and strengthens immunity (Figure 1) [2].

When nutrients are consumed by the host, such as vitamins, amino acids, and dietary fiber, intestinal bacteria assimilate the nutrients and transform them into a variety of metabolites. Short-chain fatty acids (SCFAs), biogenic amines like histamine, and other metabolites generated from amino acids, such as serotonin and gamma-aminobutyric acid (GABA), are among the results of these metabolic transformations that show biological activity in both healthy and pathological settings [2-3]. The synthesis of these substances may also lead to changes in the makeup of microorganisms (Figure 1). In the intestinal lumen, indigestible carbohydrates are fermented to produce SCFAs such as butyrate, propionate, and acetate. In addition to providing human colonic epithelial cells with metabolic energy, metabolically active SCFAs are essential for many biological functions. Furthermore, the gastrointestinal tract's resident good bacteria, primarily *Bifidobacterium* and *Lactobacillus* species, proliferate when prebiotic carbohydrates like inulin and fructo-oligosaccharides ferment [2].

**Figure 2:**
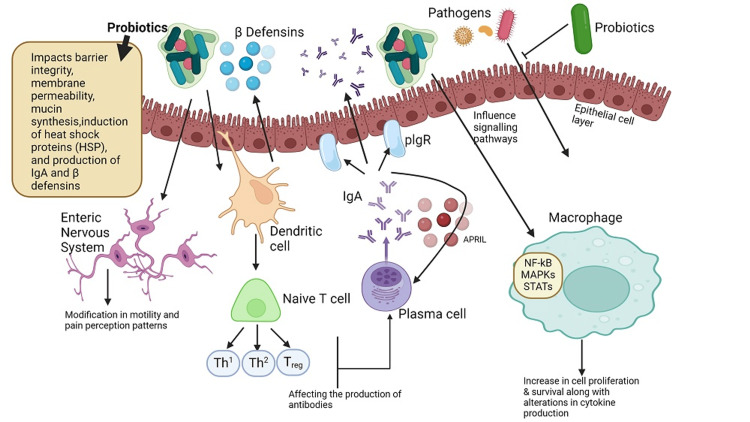
Mechanisms of probiotics in the human GI tract Probiotics have the potential to influence the composition of intestinal microbial communities and inhibit the proliferation of harmful pathogens by stimulating the host's production of β-defensin and Immunoglobulin A (IgA). Moreover, probiotics may strengthen the intestinal barrier by preserving tight junctions and promoting mucin production. Additionally, probiotics may modulate the immune system by regulating cytokine secretion through signaling pathways such as nuclear factor kappa B (NFκB) and mitogen-activated protein kinases (MAPKs), impacting the proliferation and differentiation of immune and epithelial cells. Furthermore, probiotics can regulate gut motility and pain perception by controlling pain receptor expression and neurotransmitter secretion. plgr: polymeric immunoglobulin receptor, APRIL: a proliferation-inducing ligand, STATs: signal transducer and activator of transcription proteins, Treg: regulatory T-cells, Th1: T helper cell type 1, Th2: T helper cell type 2, GI: gastrointestinal Image Credits: Created With BioRender.com

Our gut microbiota is subjected to a variety of stressors during life, such as antibiotics, poor diet, alcohol, strenuous exercise, and pathogenic microbes. Dysbiosis is the state in which gut microbiota are unable to fight off these assaults, leading to a long-lasting change that might not be conducive to health [3]. Targeted probiotics may be utilized to correct the upset gut's microbial balance and reverse dysbiosis. Probiotic agents include *Saccharomyces boulardii*, the gram-negative *Escherichia coli* strain Nissle 1917, several strains of *Lactobacilli *that produce lactic acid, and several strains of *Bifidobacterium* [4]. Numerous mechanisms are employed by probiotic microorganisms to exert their effects, including modulation of immune function, interaction with the host's resident microbiota, production of organic acids and antimicrobial compounds, enhancement of the integrity of the gut barrier, production of enzymes, elevation of the release of anti-inflammatory cytokines, stimulation of antibody secretion, and stimulation of natural killer cell activity (Figure 2). Research suggests that specific strains, dosages, and durations must be chosen and matched to the specific condition to elicit a therapeutic effect [5].

With the goal of helping to treat and prevent a variety of gastrointestinal conditions, such as antibiotic-associated diarrhea (AAD), irritable bowel syndrome with diarrhea (IBS-D), constipation, ulcerative colitis, post-surgical complications, severe illnesses, and pediatric gut health, this comprehensive review article aims to analyze and summarize the results of randomized controlled trials carried out over the past ten years on the efficacy of probiotics in modulating the microbiota of the digestive tract. This would enable healthcare providers to identify future lines of exploration by compiling the most recent data.

## Review

Antibiotic-associated diarrhea and irritable bowel syndrome with diarrhea

**Figure 3:**
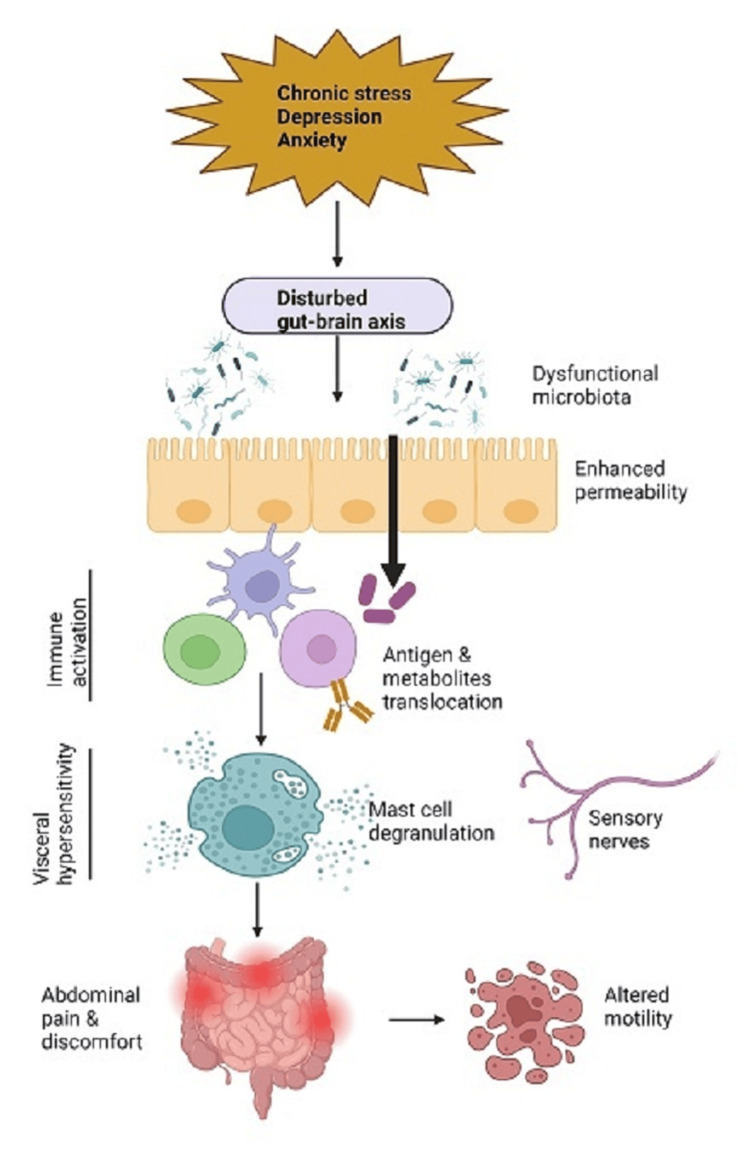
A schematic representation of the IBS pathophysiology Psychological, physiological, and neuro-gastroenterological factors are thought to be involved in the generation of irritable bowel syndrome (IBS) symptoms, including bloating, abdominal pain, and altered motility. Image Credits: Created With BioRender.com

The majority of studies conducted in the last few years have concentrated on the intricate relationship between gut microbiota, the use of antibiotics, and the incidence of diarrhea. The main causes of AAD, a common side effect of antibiotic therapy, are thought to be disruptions in metabolic pathways and intestinal dysbiosis [6]. Likewise, altered microbiota and other physiologic mechanisms like altered mobility of the gastrointestinal (GI) tract, visceral discomfort, raised intestinal permeability, immune activation, and disruptions in brain-gut function are also linked to altered microbiota and IBS, a variety of functional bowel disorders characterized primarily by abdominal discomfort and bowel habit irregularities (Figure 3) [7]. Patients are classified as having IBS-D if more than 25% of their bowel movements are consistent with a Bristol stool form score of 6 or 7.

Dietary changes, lifestyle changes, and pharmaceutical interventions such as 5-HT3 antagonists, opioid agonists, antibiotics, and bile salt sequestrants are currently accessible therapies for IBS-D [8]. These therapies may provide some comfort, but each person may respond differently, and some may have negative side effects that could be fatal. For instance, loperamide, a peripherally acting opioid receptor agonist, effectively lowers abdominal pain and enhances the frequency and quality of stools in people with IBS-D [9]. However, a retrospective study discovered a connection between ventricular dysrhythmias and long-term loperamide use [10].

There have not been many investigations done to date that explain the intricate physiological relationship between probiotics and gut microbiome colonization, despite improvements in screening methods and serologic testing. It was discovered that *Lactiplantibacillus plantarum* CCFM1143 could modulate the SCFAs, particularly acetic and propionic acids; inhibit the increase in interleukin 6 (IL-6) and the reduction in motilin; and regulate the gut microbial communities, specifically lowering the proportion of *Bacteroides* and *Eggerthella* and boosting loads of *Akkermansia, Anaerostipes,* and *Terrisporobacter* [11]. Furthermore, a study by Wieërs et al. demonstrated that *Pseudomonas* gut colonization significantly decreased from 25% to 8.3% in patients treated with a probiotic combination of *Saccharomyces boulardii*, *Lactobacillus acidophilus* NCFM, *Lactobacillus paracasei* Lpc-37, *Bifidobacterium lactis* Bl-04, and *Bifidobacterium lactis* Bi-07 (Bactiol duo®) after antibiotic therapy [12]. Additionally, the same study showed that following antibiotic therapy, the colonization of the stomach with *Enterobacteria* that produce ampicillin temporarily increased and then decreased following a probiotic intervention. Therefore, employing probiotic blends could be useful in preventing GI infections from colonizing the gut. For example, the targeted multi-strain probiotic BioKult has been shown to significantly lessen IBS-D patients' GI pain [13]. Following treatment with *Lacticaseibacillus paracasei* DG probiotics, there was a significant reduction in the fecal abundance of 13 bacterial taxa, including *Coriobacteriaceae, Dorea spp., and Collinsella aerofaciens*, among IBS-D and IBS-M patients [14]. These taxa were found to be overrepresented among probiotic responders. The administration of BIO-25 probiotic capsules was found to reduce bilophila in women with IBS-D in another placebo-controlled study. The study also suggested that patients with a more compatible baseline microbiome profile could be identified in order to customize specific probiotic formulations for individual patients, which could improve treatment responses, decrease the use of inappropriate probiotics, and lower associated healthcare expenses [15].

Probiotics are typically seen to be both reliable and beneficial in treating the symptoms experienced by IBS-D sufferers. When patients received multi-strain antibiotic treatment, two investigations found no negative side effects [13,16]. Furthermore, Yang et al. found no adverse effects among patients treated with *Lactiplantibacillus plantarum* CCFM1143 following a follow-up [11]. The effectiveness of treating IBS-D patients with combined diet modification and probiotics has only been partially studied. Patients who combined a *Bifidobacteria* probiotic along with an IgG-testing-informed elimination diet demonstrated considerable relief in their symptoms, despite the fact that their total IgG titers did not significantly decrease, according to one study that examined patients who took a placebo and continued eating an IgG-positive diet [17].

Constipation

Approximately 14% of people globally suffer from chronic idiopathic constipation, a GI condition affecting colonic or anorectal function [18]. Constipation most likely has a complex etiology and pathophysiology. The underlying pathophysiology is complicated and involves the reciprocity of different contributing factors, including physical weakness brought on by frailty, medication side effects from antiparkinsonian drugs, enteric nervous system-related GI dysfunction caused by alpha-synuclein buildup, and sphincter contractions that are reduced as a result of the disease [19]. As a result of this, treating constipation presently is still challenging, despite the fact that there are a few different approaches that may be taken, such as changing one's lifestyle and eating habits, using bulking substances, stool softening agents, stimulant laxatives, or doctors' prescription medications [20]. In the past, the only treatments for dysbiosis of the gut that altered the microbial composition of the feces and caused constipation were isoosmotic macrogol and lubiprostone, which showed excellent outcomes in terms of frequency as well as consistency of the stool [21-22]. Similar findings have been made on the basic role that GI bacteria play in gut motility. While it is evident that some probiotic strains have proven to be quite helpful in treating constipation, there is still a lot of uncertainty about the optimal way to utilize them and which strains are most effective for different scenarios and patients. Within eight weeks of probiotic treatment, there was a notable improvement in the frequency of bowel openings and gut time transit in individuals suffering from constipation, according to a placebo-controlled trial utilizing Hexbio®, a multi-strain probiotic. The weekly mean bowel movement was significantly higher in those who took the multi-strain probiotic (including *Bifidobacterium *sp. and* Lactobacillus* sp.) as opposed to the placebo. Moreover, the proportion of patients who continued to experience constipation was considerably lower in the probiotic group (using *Lactobacillus* sp. and *Bifidobacterium* sp.) (22.7%) compared to the placebo group (57.7%) [23]. In a different investigation, the effects of supplementing with two different dosages of *Bifidobacterium animalis *subsp. *lactis *HN019 for 28 days on chronic idiopathic constipation were examined [24]. Although there were no statistically significant variations in the primary or secondary outcomes across the therapies, a post hoc analysis showed that irrespective of the HN019 dosage, individuals with less than three weekly bowel movements had a significant increase in weekly bowel movement frequency. According to these results, future HN019 therapies may concentrate on addressing poor stool frequency. In a study, participants received a probiotic product containing *Lactobacillus acidophilus* DDS‐1, *Bifidobacterium animalis* subsp. *lactis* UABla‐12, *Bifidobacterium longum* UABl‐14, and *Bifidobacterium bifidum* UABb‐10 for a duration of four weeks. The majority of participants in the probiotic group reached a balanced pattern in less than a week, indicating that they had faster restoration of bowel frequency and consistency [25].

Probiotics' impact on elderly constipation was documented in two investigations. One investigation evaluated the effects of *Bifidobacterium longum* BB536 on older individuals with chronic constipation using the constipation scoring system [26]. Probiotic treatment significantly increased bowel motions, even if the primary goal of addressing changes in total score was not significant. The results of this study suggest that supplementing with *Bifidobacterium longum* BB536 is a safe and semi-effective way to help older people who suffer from persistent constipation. In another study, testing was done on a novel liquid probiotic formulation that contained *Bifidobacterium animalis* subsp. *lactis* BLC1, *Lactobacillus acidophilus* LA3, and *Lactobacillus casei* BGP93. In older patients with functional constipation, the results show effectiveness, safety, and good tolerance [27].

Major surgical procedures and severe conditions

As the frequency of elective surgeries rises, infection, morbidity, and mortality will also rise, especially in elderly patients with numerous ailments. Despite improvements in global healthcare accessibility, postoperative complications are on the rise. Infections strike 10-20% of surgery patients [28]. Treatment options for postoperative infections include wound care, surgery, and antibiotics. Adverse effects from antibiotics, scar tissue formation from surgery, and delayed healing from incorrect wound care are all possible outcomes. The gut microbiota must be varied and in balance in order to sustain homeostasis. Prolonged inflammation and a rise in carcinogenic chemicals linked to dysbiosis can have major repercussions, including colorectal cancer. Probiotics, on the other hand, preserve eubiosis and hence aid in averting such instances [29]. Probiotics increase patients' chances of a successful and risk-free surgical recovery from colorectal adenocarcinoma, according to multiple studies. Patients who took probiotics had a six-month reduction in surgical complications and mortality, according to a randomized controlled experiment [30]. Six strains of probiotics, *Lactobacillus* and *Bifidobacteria*, were investigated in a six-month clinical research to see how they affected the inflammatory cytokines and clinical outcomes of patients with colorectal cancer [31]. The administration of probiotics was demonstrated to be advantageous by the reduction of pro-inflammatory cytokine levels (TNF-α, IL-6, IL-10, IL-12, IL-17A, IL-17C, and IL-22). In a different randomized, double-blind study, digestive enzymes (Aczym) or probiotics (*Clostridium butyricum* or *Bifidobacterium longum*) reduced gas, malodorous flatulence, burping, burning sensations, abdominal noises, and abdominal cramping in sixty patients who had gastric bypass surgery due to extreme obesity and experienced GI symptoms following the procedure [32]. These findings suggest that probiotics or digestive enzymes may help with post-gastric bypass discomfort.

However, research has shown that probiotics have no therapeutic effects on a number of different illnesses. In a double-blind, randomized, placebo-controlled research, the effects of *Lactiplantibacillus plantarum* 299v (Lp299v) supplementation on nutritional status, enteral formula tolerance, and quality of life were investigated in cancer patients undergoing home enteral nutrition. There was a significant increase in serum albumin levels when comparing Lp299v versus placebo. Furthermore, Lp299v reduced the amount of vomiting and flatulence. Interestingly, though, neither group's quality of life improved statistically, suggesting that enteral nutrition rather than Lp299v supplementation was the cause [33]. Probiotics and immune nutrients were integrated into enteral ecoimmunonutrition, which was assessed in connection with the findings of a study on patients with stomach cancer. On the seventh postoperative day after surgery, the ecoimmunonutrition group had greater CD4+ concentrations and lower C-reactive protein levels, but there were no appreciable variations in nutritional status or problems between the groups. A shorter time to first flatus in this group than with enteral feeding suggests improvements in intestinal healing and immunological function [34]. Another study examined the impact of preoperative *Saccharomyces* *boulardii* probiotic medication on intestinal mucosal inflammatory cytokine levels in colon surgery patients. Probiotic-treated patients showed significantly lower levels of mRNA for IL-1β, IL-10, and IL-23A than did the control group. Regarding cytokine mRNAs, probiotics clearly had no effect on infection rates, even if they may have decreased the expression of intestinal mucosal inflammatory cytokines. Both groups had comparable levels of infection and other cytokine mRNA following surgery [35].

Ulcerative colitis

The gut microbiome is impacted by ulcerative colitis (UC) in a number of ways. A noteworthy feature is the decline in microbial diversity, which is attributed to a rise in potentially hazardous bacteria like *Proteobacteria* and a fall in beneficial microbes like *Firmicutes* and *Bacteroidetes *[36]. Additionally, some bacterial species have an unbalanced makeup, leading to an overabundance of pro-inflammatory microorganisms. Furthermore, the mucosal barrier's integrity is damaged, which permits germs to infiltrate the gut wall's deeper layers and cause inflammation. The immune system's response exacerbates the dysbiosis further, perpetuating the cycle of inflammation and microbial disruption [37-39]. The hazards and issues associated with UC vary in severity depending on the individual; the most important of them is the heightened risk of colorectal cancer [40]. Severe inflammation may also have other risks, such as poor nutrition absorption, toxic megacolon, and extraintestinal symptoms. Numerous studies have examined the modulatory influence of probiotics on the gut microbiota in UC, finding intricate interactions that may lead to novel therapeutic strategies. According to Yilmaz et al., eating fermented milk products like kefir modifies the gut microbiota, as evidenced by changes in the bacterial load of *Lactobacillus* found in the feces [37]. Several investigations have also elucidated the advantages associated with the application of probiotic blends in the management of UC. Giving a combination of six probiotic strains to people with UC may have a number of short-term benefits, per a research by Sanchez-Morales et al. [41]. When compared to the control group, this study demonstrated that patients who received a combination of six probiotic strains showed significant improvements in histological findings and a trend toward improvement in disease activity. Additionally, the study showed that probiotic therapy was associated with better eating tolerance in individuals with UC. According to this, probiotics may aid in enhancing digestive tolerance, which may have an effect on an individual's overall nutritional status and general well-being. A third study found that probiotic pills with the WeChat platform may help patients with mild-to-moderate UC manage their health in terms of their general quality of life, inflammatory markers, and nutritional status [39]. Furthermore, a study by Palumbo et al. indicates that probiotic mix (*Lactobacillus salivarius*, *Lactobacillus acidophilus*, and *Bifidobacterium bifidus* strain BGN4) and mesalazine administered over an extended period of time may be an alternative to corticosteroids for the treatment of mild-to-moderate UC and be associated with long-lasting beneficial effects and improved clinical response [36]. Additionally, it was discovered that when used as an adjuvant therapy to 5-aminosalicylic acid, the probiotic Mutaflor (EcN) improves clinical responses, increases rates of endoscopic remission, and prevents exacerbations of the Inflammatory Bowel Disease Questionnaire score in patients with mild-to-moderate UC [42]. Finally, the multi-strain probiotic Symprove^TM^ has positive effects, especially when it comes to reducing GI inflammation in asymptomatic UC patients who do not exhibit any symptoms. Post-hoc analyses showed fecal calprotectin levels were significantly (p<0.015) reduced in the UC patients receiving the Symprove^TM ^probiotic as opposed to placebo [43]. However, *Bifidobacterium breve* strain Yakult (BFM) fermented milk did not show a significant benefit in preserving remission in patients with quiescent UC, and the study was discontinued due to a lack of effectiveness of treatment. The study involved Japanese patients who were randomized to receive either BFM fermented milk containing *Bifidobacterium breve* strain Yakult and *Lactobacillus acidophilus *or a matching placebo for 48 weeks [44]. Probiotics as a primary or stand-alone treatment for UC are not yet supported by sufficient evidence, despite some studies demonstrating encouraging outcomes.

Pediatric GI health

Since the infant microbiota is known to be dependent on a number of variables, including breastfeeding, the mode of birth, the use of antibiotics, and the introduction of new foods, it is expected that it will change throughout the first year of life. Functional GI diseases such as newborn colic, regurgitation, functional constipation, and functional diarrhea are more common at this time. As one of the main symptoms of dysbiosis, pain is where probiotics come into play. Probiotics are essential for maintaining children's GI health, which emphasizes their importance as a mainstay of pediatric therapy. Pain is one of the main symptoms of dysbiosis, and probiotics have shown promise in the management of intestinal pain and inflammation in children [45]. These disorders are commonly encountered in clinical practice. Regardless of breastfeeding or formula feeding, inconsolable weeping and irritation are the hallmarks of infant colic, the condition that is the main cause of consultation for babies. Two strains of probiotics obtained from breast milk (*Bifidobacterium breve* CECT7263 and *Lactobacillus fermentum* CECT5716) were administered to infants in a randomized, double-blind controlled study in place of simethicone. The results showed a significant improvement with probiotic administration, reducing crying time from the first week of administration. Parents also reported higher-quality sleep and reduced distress related to the disease, which supports earlier research showing the beneficial effects of probiotic medication [46]. The effectiveness of giving probiotics to a pregnant woman in the final trimester of the pregnancy is being investigated even in the perinatal stage, with the goal of determining how the baby would be affected by breast milk. The incidence of GI symptoms in newborns fed breast milk or infant formula was found to be reduced by an increase in secretory IgA and a decrease in lactoferrin, according to this double-blind, randomized trial that assessed newborn bowel movements and specifically measured levels of IgA and lactoferrin [47].

Probiotics are considered one of the therapy choices for functional constipation, one of the most prevalent conditions in the pediatric age group with multiple etiologies. *Lactobacillus reuteri *DSM17938 was used in a prospective double-blind study in place of a magnesium laxative. It showed promise by increasing the frequency of bowel movements but not their consistency, offering a treatment option that circumvents the negative effects of laxative use [48]. Probiotic supplementation has been studied in premature infants, where poor colonization of the intestinal microbiome and other factors can lead to necrotizing enterocolitis. The results show improvement in the neonate's overall conditions, including reduced regurgitation, shorter feeding times, early feeding tolerance, increased anthropometric measurements, and increased intestinal motility. These improvements prevent the onset of necrotizing enterocolitis and shorten hospital stays [49-51]. Patients with celiac disease who took *Bifidobacterium breve *supplements for three months showed a significant decrease in TNF-a and an increase in *lactobacilli *species that have anti-inflammatory properties [52].

In contrast, administering probiotics to patients with necrotizing enterocolitis grades 2 and 3 showed no changes once the disease was established [49]. Furthermore, when dealing with immunocompromised or very low birth weight (<1000 g) patients, several studies advise against using probiotics. One of the most common reasons for hospitalization in adults and children is infectious gastroenteritis. Probiotics are recommended and approved by a number of guidelines; however, the data is conflicting since multiple studies have not demonstrated statistically significant benefits in lowering the duration of diarrhea or the severity of the disease when compared to supportive interventions [53].

Table 1 shows the selected RCTs of probiotics for various GI conditions. Additionally, an increasing number of studies have explored the role of diverse probiotic combinations in GI disorders (Table 2).

**Table 1:** Selected RCTs of probiotics for various GI conditions RCT: randomized controlled trial, CFU: colony forming unit, GI: gastrointestinal

Study	Study Design	n (Number of Patients)/Age	Regimen	Duration	Outcomes
Ishaque et al., 2018 [13]	RCT	400 (18-55 years)	Multistrain probiotic (BioKult): 14 different bacterial strains. 2 capsules daily (2 billion CFUs/capsule)	16 weeks	The number of bowel motions per day from month two onwards was significantly reduced in the probiotic group thereby improving overall quality of life
Majeed et al., 2016 [16]	RCT	36 (18-55 years)	*Bacillus coagulans* MTCC5856 tablet containing 2x10^9 ^CFU/day	13 weeks	Significant decrease in clinical symptoms like bloating, vomiting, diarrhea, abdominal pain, and stool frequency in a patient group receiving the probiotic
Ibarra et al., 2018 [24]	RCT	228 ( 18-70 years)	*Bifidobacterium animalis* Subsp*. lactis* HN019 at two doses: 1x10^10 ^CFU and 1x10^9 ^CFU	4 weeks	HN019 was well tolerated at high and low doses and improved bowel motion frequency in adults with constipation
Martoni et al., 2019 [25]	RCT	94 (18-65 years)	Multistrain probiotic *Lactobacillus acidophilus* and three *Bifidobacterium* species (1.5x10 (10)) CFU/day	4 weeks	The probiotic group showed a faster normalization of stool frequency and consistency
Kubota et al., 2020 [48]	RCT	60 (6 months-6 years)	Probiotic *Lactobacillus reuteri *DSM17938 either alone or in combination with magnesium oxide	4 weeks	Both treatments were effective in the management of functional constipation in young children when compared with those receiving placebo
Cui et al., 2019 [49]	RCT	114 (30 weeks-37 weeks)	*Limosilactobacillus reuteri* DSM17938 ( 1x10^8 ^CFU): 5 drops once daily	Until discharge (minimum duration 7 days)	*Limosilactobacillus reuteri* may be a useful probiotic for improving early feeding tolerance in preterm infants, promoting growth, and increasing the frequency of defecation

**Table 2:** Selected studies on probiotic formulations IBS-D: irritable bowel syndrome with diarrhea, UC: ulcerative colitis

Study	Probiotics Formulation	Effective Against
Abdellah et al., 2023 [54]	Bifidobacterium lactis, Lactobacillus acidophilus, Lactobacillus plantarum, Lactobacillus sativaius	IBS-D
Bajramagic et al., 2019 [30]	Lactobacillus acidophilus, Lactobacillus plantarum, Lactobacillus casei, Lactobacillus rhamnosus, Bifidobacterium lactis, Bifidobacterium bifidum, Bifidobacterium, breve, Streptococcus thermophilus	Colorectal carcinoma
Baldassarre et al., 2016 [47]	Lactobacillus paracasei, Lactobacillus acidophilus, Lactobacillus plantarum, Lactobacilus delbrueckii subsp. bulgaricus, Bifidobacterium longum, Bifidobacterium breve, Bifidobacterium infantis, streptococcus thermophilus	Infantile colic
Barker et al., 2017 [55]	Lactobacillus paracasei, Lactobacillus acidophilus, Bifidobacterium lactis	Clostridium difficile-induced diarrhea
Bjarnason et al., 2019 [43]	Lactobaillus rhamnosus, Lactobacillus plantarum, Lactobacillus acidophilus, Enterococcus faecium	UC and Crohn's disease
Evans et al., 2016 [56]	Lactobacillus helveticus, Lactobacillus rhamnosus	Antibiotic-associated diarrhea
Freedman et al., 2020 [52]	Lactobacillus helveticus, Lactobacillus rhamnosus	Acute gastroenteritis in children under five years of age.
Hod et al., 2018 [15]	Lactobacillus rhamnosus, Lactobacillus casei, Lactobacillus paracasei, Lactobacillus plantarum, Lactobacillus acidophilus, Bifidobacterium bifidum, Bifidobacterium longum, Bifidobacterium breve, Bifidobacterium infantis, Streptococcus thermophilus, Lactobacillus vulgaricus, Lactobacillus lactis	IBS-D
Labenz et al., 2022 [57]	Bifidobacterium bifidum, Bifidobacterium lactis, Enterococcus rhamnosus, Lactococcus lactis	Colon cancer

## Conclusions

The most recent studies on the advantages of probiotics for GI health and their treatment of various GI disorders are compiled in this review. Probiotics have demonstrated promise in controlling the gut microbiota and reducing GI disease symptoms, such as UC, diarrhea brought on by antibiotics, and other GI disorders. While some studies show clear benefits, other research shows contradictory results or no effect at all. The variation in probiotic strains, dosages, and study populations emphasizes the need for more research to optimize the efficacy and uniformity of probiotic therapy. Despite the gaps and problems that now exist, probiotics remain a valuable adjuvant medicine in the treatment of GI disorders, offering a potentially safe and effective means of improving patients' quality of life. To fully achieve the therapeutic potential of probiotic therapy in GI health, further study is needed to understand the probiotics' mechanisms of action, optimal formulations, and patient-specific responses.
